# Bioequivalence assessment of imeglimin hydrochloride tablet, a novel glimin-class antidiabetic agent

**DOI:** 10.1007/s00210-025-04780-x

**Published:** 2025-11-26

**Authors:** Mamdouh R. Rezk, Kamal A. Badr, Ahmed Abd El Hamid Hosny, Aya M. AbdelMagid

**Affiliations:** 1 Pharmaceutical Analytical Chemistry Department, Faculty of Pharmacy, Cairo University, Cairo, Egypt; 2 Advanced Research Center (ARC), Nasr City, Cairo, Egypt; 3 Pharmaceutics Department, Faculty of Pharmacy, Deraya University, New Minya, Egypt; 4 Atco Pharmaceutical Company, R&D Department, Cairo, Egypt; 5 Clinical Pharmacy Department, Faculty of Pharmacy, Cairo University, P.O. Box: 11562, Cairo, Egypt

**Keywords:** Imeglimin, Bioequivalence, Tablet, Diabetes, Cost-saving

## Abstract

**Purpose:**

While imeglimin’s, oral antidiabetic agent, efficacy and safety are established in Japanese and Western populations, pharmacokinetic data from Middle Eastern and African cohorts remain limited. This study aimed to evaluate the bioequivalence of test and reference imeglimin formulations under fasting conditions in healthy Egyptian adults.

**Methods:**

In a randomized, open-label, two-period, two-sequence crossover trial, healthy male and female adults received a single 500 mg oral dose of either formulation under fasting conditions, separated by a one-week washout. Adverse events (AEs) were assessed via direct questioning, participants’ reporting, vital signs monitoring, and clinical laboratory evaluations. Plasma concentrations were measured up to 72 h post-dose, and pharmacokinetic parameters were derived using non-compartmental analysis. Bioequivalence was concluded if the geometric mean ratios (GMRs) and 90% confidence intervals (CIs) for peak plasma concentration (C_max_), area under the concentration–time curve to 72 h (AUC_0-72_), and to infinity (AUC_0–inf_) were within 80–125%.

**Results:**

In 29 healthy participants, all pharmacokinetic endpoints met bioequivalence criteria. GMR (90% CI) for each pharmacokinetic endpoint were: C_max_ 101.75% (94.42–109.64); AUC_0–72_ 100.66% (94.28–107.46); AUC_0–inf_ 100.13% (93.77–106.92). The intrasubject variability was low (14–17%). Both formulations were well tolerated, with only mild transient adverse events (headache, abdominal pain) and no serious events.

**Conclusion:**

The test formulation of imeglimin was bioequivalent to the reference, with a favorable safety profile. This first pharmacokinetic and bioequivalence study in a Middle Eastern/African population supports regulatory approval and enhances opportunities for affordable access to imeglimin in type 2 diabetes management.

Clinical trial number.

The ClinicalTrials.gov registration number is NCT07127094, retrospectively registered on August 15, 2025.

**Graphical Abstract:**

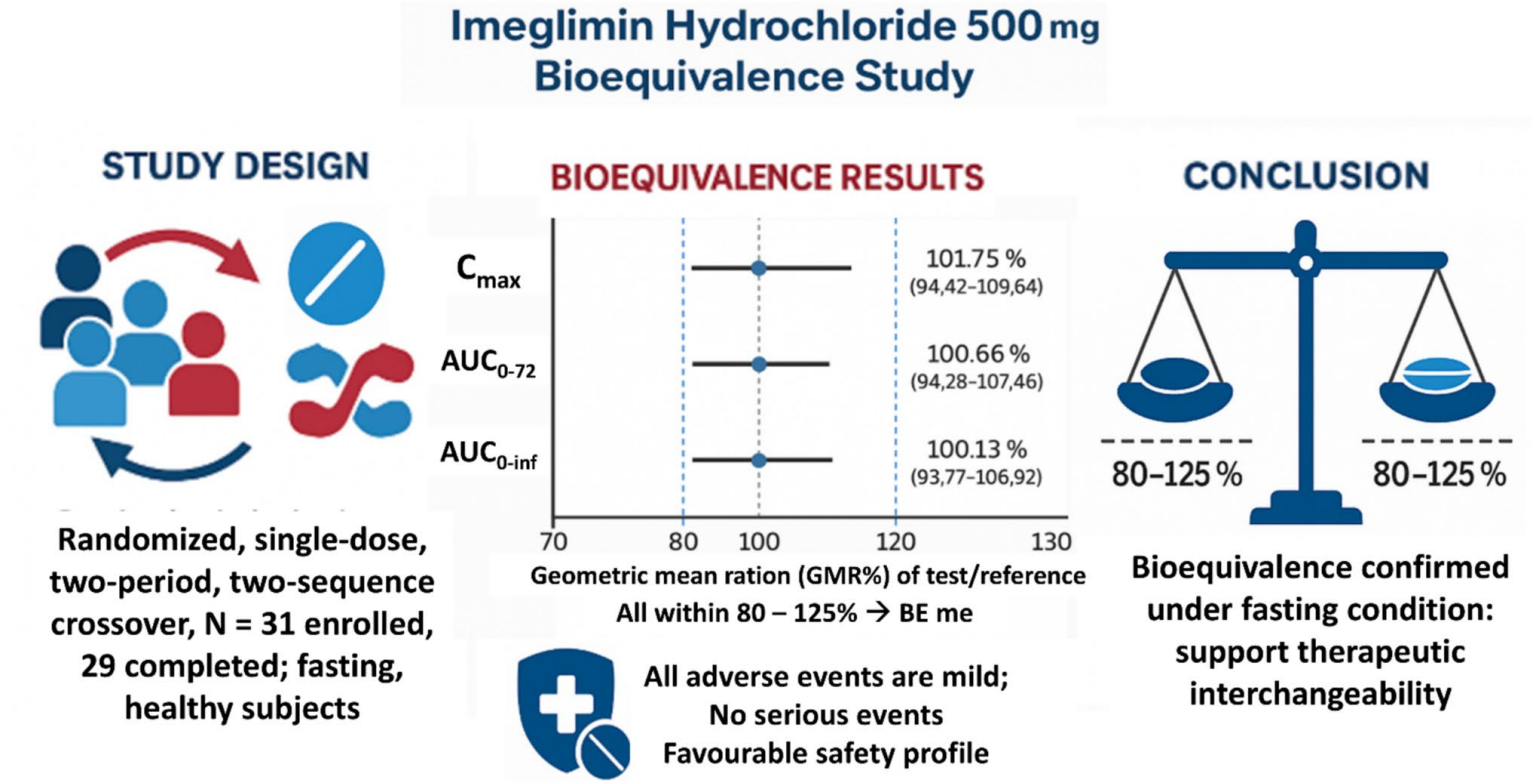

## Introduction

Diabetes mellitus (DM) is a major global health burden and one of the top ten causes of death worldwide (WHO (2024)). Type 2 diabetes (T2D) represents over 95% of all diabetes cases and is rising rapidly, particularly in low- and middle-income countries (LMICs). Despite therapeutic advances, achieving durable glycemic control remains suboptimal due to drug-related adverse effects, and adherence challenges (Giruzzi 2021).

Imeglimin, a first-in-class oral agent from the glimin family, offers a novel therapeutic approach for T2D by improving mitochondrial bioenergetics. It enhances glucose-dependent insulin secretion, preserves β-cell function, and improves insulin sensitivity in muscle and liver (Vuylsteke et al. 2015; Konkwo and Perry 2021; Fouqueray et al. 2022). Approved in Japan in 2021 for T2D under its International Nonproprietary Name (INN), imeglimin, though not yet U.S. Food and Drug Administration (FDA) approved, represents a promising therapeutic option to global T2D management (Lamb 2021).

In phase III TIMES program conducted in Japanese adults with T2D, imeglimin 1000 mg twice daily demonstrated consistent efficacy and safety across various therapeutic settings. As monotherapy and add-on therapy to insulin or other oral agents, imeglimin produced clinically meaningful and sustained HbA1c reductions with favorable safety across the phase III TIMES program (Dubourg et al. 2021b; Dubourg et al. 2022; Reilhac et al. 2022). Imeglimin is well tolerated, with mostly mild gastrointestinal symptoms, favorable cardiovascular safety profile and low risk of hypoglycemia with monotherapy but occurred more frequently when combined with sulfonylureas or insulin (Dubourg et al. 2020; Dubourg et al. 2022; Dubourg et al. 2021b; Reilhac et al. 2022).

Following a single 1000 mg oral dose, imeglimin reaches peak plasma concentrations in 3.5 h, with steady state achieved by day 5. Plasma protein binding is minimal (~ 5–6%) and the apparent half-life ranges from 9 to 20 h, typically around 13 h at therapeutic doses. Imeglimin undergoes negligible metabolism and is primarily eliminated unchanged in urine, with renal clearance averaging 26–39 L/h, indicating active tubular secretion (Clémence et al. 2020; Fouqueray et al. 2022). Hepatic impairment produces modest, non-clinically significant exposure increases; however, its use is not recommended in patients with severe liver dysfunction or an estimated glomerular filtration rate (eGFR) < 45 mL/min/1.73 m^2^ (Clémence et al. 2020; Chevalier et al. 2021; Lamb 2021).

Given the growing global burden of T2D, improving access to durable and effective therapies is a public health priority (Rodríguez-Gutiérrez et al. 2021). With its unique mechanism of action, favorable tolerability, and potential for end-organ protection, imeglimin is a promising option for T2D management. However, published data on its pharmacokinetics (PK) are limited to Caucasian and Japanese participants, highlighting the need for evaluation in other populations. Glimcoza is a locally manufactured 500 mg imeglimin hydrochloride formulation developed for the Egyptian market, while Twymeeg® (Sumitomo Dainippon Pharma Co., Ltd., Japan) serves as the reference product. Therefore, demonstrating bioequivalence (BE) between Glimcoza and Twymeeg is both clinically and regulatorily important to ensure therapeutic interchangeability, support local regulatory approval, and expand access to this novel antidiabetic therapy in Egypt. Accordingly, this study aimed to evaluate the BE and safety of Glimcoza versus Twymeeg under fasting conditions in healthy adult participants.

## Materials and methods

### Study setting and ethical consideration

The BE study was conducted at the Advanced Research Center (ARC), Cairo, Egypt, between March and July 2025. Ethical approval was granted by the ARC Independent Ethics Committee on March 9, 2025 (IEC No.: IEC_060325_01). The trial was retrospectively registered at ClinicalTrials.gov NCT07127094 on August 15, 2025. All study procedures adhered to the ethical principles of the institutional ethics committee, the International Conference on Harmonization’s Good Clinical Practice (ICH-GCP) Guidelines (TGA (2018)), and the Declaration of Helsinki (1964) (World Medical Association 2013) and its later amendments or equivalent standards. Written informed consent was obtained from each participant before any study-related activity, following clear explanation of the study’s objectives, procedures, potential benefits, and risks to ensure complete understanding of the study. All biological samples and collected data were used exclusively for study purposes, and confidentiality was strictly maintained.

### Study design and procedures

This single-center, open-label, randomized, single-dose, two-sequence, two-period crossover BE study was conducted under fasting conditions in healthy participants. Fasting conditions were selected according to regulatory guidelines, as imeglimin can be administered with or without food. This is supported by the product’s labeling information and pharmacokinetic data showing no clinically significant food effects (Lamb 2021; Poxel 2021; Fouqueray et al. 2022; EDA (2023); FDA (2024)). The study comprised pre-randomization phase (screening) and randomization phase (two study periods). The pre-randomization phase consisted of a screening period started from (Day –30) to (Day –3) with a baseline assessment on (Day –1), the day prior dosing in the first treatment period.

**Fig. 1:**
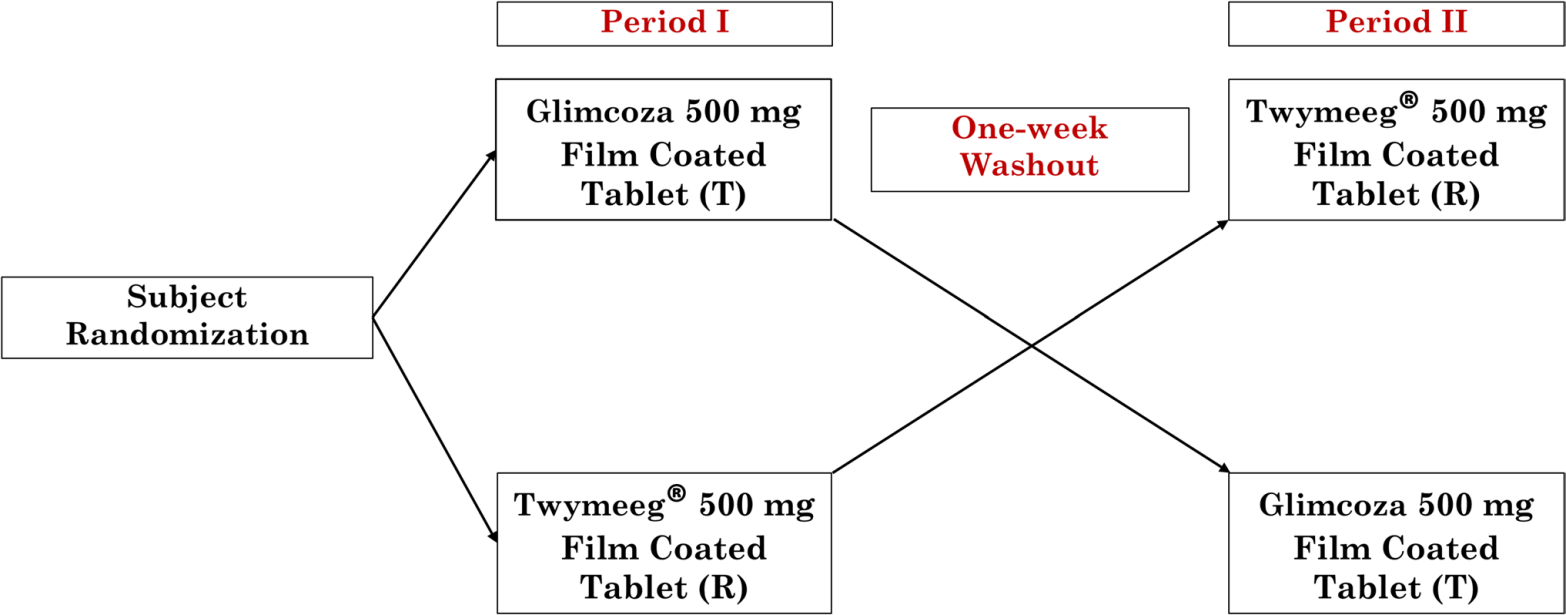
Summary of imeglimin hydrochloride oral tablet (500 mg) bioequivalence study periods

At the screening visit, assessments were performed including medical history, physical examination, vital signs, a 12-lead ECG, and anthropometric measurements (including height and weight, which were recorded using calibrated equipment to calculate BMI, which served as part of the eligibility assessment). Laboratory tests were performed including complete blood count, clinical chemistry, lipid profile, urinalysis, fasting blood glucose, pregnancy screening (for females only), and serology for HIV, hepatitis B surface antigen (HBsAg), and hepatitis C immunoglobulin G (HCV IgG). In accordance with the Egyptian Guidelines for Conducting Bioequivalence Studies (EDA (2023)), urine drug screening was performed to exclude participants with a history of drug abuse. Screening included tests for amphetamines, barbiturates, benzodiazepines, cannabinoids, cocaine, opiates, and tramadol. Only participants with results within normal limits or with clinically non-significant deviations were eligible for the randomization phase. This randomization phase included two treatment periods separated by a one-week washout to prevent carryover effects. Participants were randomly assigned in a 1:1 ratio to receive a single oral tablet of either the test product (Glimcoza) or reference product (Twymeeg®) after a minimum 10-h fast, following a TR-RT crossover sequence (Fig. 1). The randomization schedule was generated using R statistical software (version 4.2.1, R Foundation for Statistical Computing, Vienna, Austria) according to the planned sample size in the study protocol, considering the number of treatments and the crossover study design.

Following a 10-h overnight fast, a single 500 mg imeglimin hydrochloride tablet (test or reference formulation based on randomization plan) was administered orally with 240 mL of water. Fluid intake was prohibited for one hour before and after dosing. For consistency among all participants, all of them received a standardized diet, with meals and beverages served at the same timelines in each period in compliance with regulatory guidelines (EDA (2023); FDA (2024)). Two meals were served to the participants during their stay in the study center at 4- and 8-h post-dose. The first and second meals provided approximately 850–900 kcal and 700–750 kcal, respectively, with a comparable macronutrient distribution of approximately 25% protein, 45–50% carbohydrates, and 30% fat. Both were prepared using locally available ingredients, reflecting typical Egyptian dietary patterns, and were designed to ensure adequate energy intake. The investigational products were given at the trial site under the supervision of the clinical study team, followed by mouth and hand checks to ensure compliance.

On Day 11, a follow-up visit was conducted for all enrolled participants, during which a physical examination and vital sign assessment were performed, participants were queried regarding any experienced adverse events, and a complete blood count (CBC) was obtained.

### Eligibility criteria

The study included healthy adult volunteers who met the following criteria.

### Inclusion criteria

Healthy participants from both genders (male and female) aged 18—55 years with a calculated body mass index (BMI) between 18.5 and 30 kg/m^2^ who were willing and able to comply with all study requirements. The eligibility criteria required participants to be in good overall health, with no clinically significant diseases or conditions as determined by the principal investigator based on medical history, physical and anthropometric examinations, vital signs, a baseline 12-lead electrocardiogram (ECG) conducted during screening, and routine laboratory testing. All participants were required to have negative screening results for human immunodeficiency virus (HIV), hepatitis B and C, as well as a negative urine drug abuse screen. Female participants were required to have effective contraception throughout the study and for at least 30 days after study completion.

#### Exclusion criteria

Participants were excluded if they had known hypersensitivity to imeglimin or any excipient of the investigational products; clinical or laboratory evidence of organ dysfunction or serious illnesses that could affect study outcomes; clinically significant abnormal laboratory test results; a history of drug or alcohol abuse or heavy smoking; were pregnant or breastfeeding; had used of systemic medications within two weeks or within six elimination half-lives of the drug (whichever was longer); or had donated over 400 mL of blood or participated in another clinical study within two months prior first dosing.

### Materials

#### Chemicals and reagents

Imeglimin hydrochloride (Batch No. PD/BATP/22001) was obtained from Metrochem API Private Limited (Telangana, India) with a certified potency of 99.20% and a water content of 0.27%. The internal standard, Imeglimin-d6 (Batch No. SP-0521–008), was supplied by Simson Pharma Limited (Mumbai, India) with a certified purity of 99.77% according to the supplier’s certificate of analysis. Normal and hemolyzed human plasma were procured from the National Institute of Urology and Nephrology (Cairo, Egypt). All solvents and reagents used were of HPLC grade. Acetonitrile, ammonium formate, and methanol were purchased from Sigma-Aldrich (Darmstadt, Germany). Formic acid was obtained from Fisher Scientific (Pennsylvania, USA), and heparin sodium solution (5000 I.U/mL) was supplied by CID Company (Cairo, Egypt).

#### Investigational drug products

In this study, the test product was Glimcoza containing 500 mg imeglimin hydrochloride in film-coated tablet (batch no. 243193, expiration date: October 2026), produced by Atco Pharma for Pharmaceutical Industries, Egypt. The reference product was Twymeeg® (imeglimin hydrochloride 500 mg) tablet (batch no. 1519 C, expiration date: May 2026), manufactured by Sumitomo Dainippon Pharma Co., Ltd, Japan. In vitro testing was conducted, before starting the clinical phase, for both test and reference products using the specified batches, confirming their pharmaceutical equivalence.

### Pharmacokinetic analysis

In each of the two study periods, 22 blood samples (5 mL each) were withdrawn from all participants into EDTA-treated tubes according to the following sampling scheme: pre-dose (t = 0), and at 0.25, 0.5, 0.75, 1, 1.25, 1.5, 1.75, 2, 2.5, 3, 3.5, 4, 5, 6, 8, 10, and 12 (Day 1), 24, 36 (Day 2), 48 (Day 3), and 72 h (Day 4) post-dose. Any deviations from the nominal sampling times were accurately documented, and all PK calculations were performed based on the actual sampling time. A cannula inserted into a forearm vein was used to withdraw blood samples up to 12 h post-dose. Samples were collected via venipuncture at 24-, 36-, 48-, and 72-h intervals. All samples were immediately centrifuged following their collection for 5 min at 3500 rpm and 4 °C using a PRO-analytical centrifuge (Centurion Scientific Limited-PRO-Analytical CR2000, PRO Analytical Cooling, Chichester, UK). Plasma samples were transferred into labeled plastic tubes and stored at −80°C at the trial site pending PK analysis.

Plasma samples were analyzed at ARC laboratories, Cairo, Egypt, using liquid chromatography coupled with tandem mass spectrometry (LC–MS/MS), the Acquity UPLC/XEVO TQD Triple Quadrupole Mass Spectrometer (Xevo TQD, Water Corporations, Dublin, Ireland). Electrospray ionization was operated in the positive mode under multiple-reaction monitoring (MRM) conditions optimized for imeglimin and its internal standard (Imeglimin-d6). Chromatographic separation was achieved using a hydrophilic interaction column with a mobile phase of aqueous buffer and acetonitrile at a controlled flow rate. The method demonstrated linear calibration curves over a concentration range of 10 to 3000 ng/mL for imeglimin. The method was validated in line with the FDA’s bioanalytical method validation guidance, confirming compliance with parameters such as selectivity, precision, accuracy, linearity, calibration curve performance, stability, and carry-over (Zimmer 2014; FDA (2018)).

Non-compartmental PK analysis was performed using Phoenix WinNonlin version 8.3.4 (Certara USA, Inc., Princeton, NJ, USA) to calculate the primary and secondary PK parameters. Primary PK outcomes, reflecting the rate and extent of drug absorption, included the peak plasma concentration (C_max_), area under the plasma concentration–time curve from time zero to 72 h (AUC_0–72_), and AUC up to infinity (AUC_0-inf_) (FDA (2021b)). The AUC was calculated using the linear trapezoidal rule, and AUC_0-inf_ was determined as the sum of AUC_0-t_ and the extrapolated area from the last measurable concentration (C_t_) to infinity (Gabrielsson and Weiner 2012). The time to reach maximum concentration (T_max_), terminal elimination half-life (t_₁/₂_), and elimination rate constant (k_el_) were the secondary PK outcomes. The k_el_ was calculated using the slope of the log-transformed plasma concentration–time curve, and the half-life was calculated using the formula t_₁/₂_ = ln (2)/k_el_ (Gabrielsson and Weiner 2012).

### Bioequivalence evaluation

This study primarily aimed to evaluate the BE between the test and reference products in terms of the rate and extent of absorption as per FDA bioequivalence guidelines (FDA (2024)). The key PK parameters used to determine dose comparability were systemic exposure metrics, specifically AUC_0-inf_, AUC_0–72_, and C_max_ (FDA (2021a)). T_max_ is represented as median and range. Both the arithmetic mean, and the geometric mean (following log-transformation of data) were reported for all other PK parameters. The geometric mean ratio (GMR%) and corresponding 90% confidence intervals (CIs) were calculated for AUC_0-inf_, AUC_0–72_ and C_max_ to compare the dose of the test and reference products. The two formulations were considered bioequivalent if the 90% CI for the GMR% of AUC_0-inf_, AUC_0–72_ and C_max_ fell within the accepted BE boundaries of 80% to 125% (FDA (2021a), 2024).

### Safety evaluation

All participants were closely monitored throughout the study period to ensure the safety and tolerability of the study drugs. The principal investigator asked the participants whether they experienced any adverse events on an hourly basis. Moreover, the participants were instructed to immediately report any unfavorable adverse events experienced during the study period. Vital signs, including systolic and diastolic blood pressure, heart rate, respiratory rate, and body temperature, were recorded at baseline (pre-dose) and at 2-, 4-, 6-, 12-, and 72-h intervals after dosing. At the end of the study, a complete blood count was performed for all enrolled participants for follow-up. Any reported or observed adverse events were documented and evaluated by the principal investigator evaluated to assess its seriousness, severity (categorized as mild, moderate, or severe), and its potential relationship to the study drug.

### Sample size

As no published data were available on the intrasubject variability (ISV) of imeglimin hydrochloride, the sample size was determined according to the Egyptian Drug Authority Guidelines for Conducting Bioequivalence Studies. These guidelines recommend enrolling no fewer than 24 participants and adopting a two-stage sequential approach when variability is unknown (EDA (2021)). Accordingly, the planned sample size for this study was set at 31 healthy male and female participants to maintain adequate statistical power (≥ 80%) for bioequivalence determination while accounting for potential dropouts or withdrawals. If BE is met, the study can be terminated. If not and the statistical power of the completed study is below 80%, a second stage will be conducted. The number of additional subjects will be determined based on the observed ISV from the first stage of the study, assuming a test/reference ratio (T/R) of 0.95 and significance level (α) of 0.0294.

### Statistical analysis

Statistical analysis was conducted using R statistical software (version 4.2.1, R Foundation for Statistical Computing, Vienna, Austria). The BE assessment employed two one-sided t-tests, with 90% CI calculated for the log-transformed PK parameters of imeglimin hydrochloride to compare the test and reference products. The nonparametric Wilcoxon signed-rank test was used for T_max_ comparisons between the two products, with a statistical significance set at P < 0.05. To evaluate the sequence, treatment, and period effects, an Analysis of Variance (ANOVA) test was conducted on the primary PK parameters, AUC_0-inf_, AUC_0–72_ and C_max_ and their log-transformed values for examination of potential variations related to the sequence of administration, the treatment received, and the specific study period. The ISV was expressed as the coefficient of variation (CV%).

## Results

### Study population

Screening of a total of 48 participants was performed, of whom 17 participants did not meet the eligibility criteria due to reasons such as anthropometric measurements outside the acceptable protocol-defined range, positive drug tests, abnormal clinically significant laboratory results, refusal to sign informed consent, or inadequate compliance during screening phase (e.g. missed visits, participated in another study during screening phase or failure to follow study instructions). The remaining 31 participants were successfully enrolled and randomized in the study. Of these, 29 participants completed the study; one participant withdrew before dosing in the first treatment period, and another one withdrew before dosing in the second treatment period. The enrolled participants included both males and females (23 males and 8 females who were neither pregnant nor lactating). The mean age was 33.13 ± 10.16 years, with a mean height of 169.77 ± 7.12 cm, mean body weight of 74.34 ± 10.94 kg, and a mean BMI of 25.8 ± 3.51 kg/m^2^. The demographic features and baseline characteristics of the enrolled study participants are detailed in Table 1.

**Table 1:** Summary of demographics and baseline laboratory parameters of enrolled participants

Parameter (unit)	Value	Parameter (unit)	Value
Age (years)	33.13 ± 10.16	AST (U/L)	21.9 ± 7.41
Gender (n, male)	23 (73.9%)	Blood Urea (mg/dL)	24.16 ± 7.93
Height (cm)	169.77 ± 7.12	Creatinine (mg/dL)	0.71 ± 0.16
Weight (kg)	74.34 ± 10.94	Sodium (mEq/L)	140.55 ± 1.88
BMI (kg/m^2^)	25.8 ± 3.51	Potassium (mEq/L)	4.68 ± 0.43
Hb (g/dl)	13.96 ± 1.46	Total Bilirubin (mg/dL)	0.41 ± 0.19
RBCs (10^6^/mm^3^)	5.09 ± 0.45	ALP (U/L)	61.71 ± 18.48
Hct (%)	42.48 ± 3.97	Total Protein (g/dL)	7.21 ± 0.39
Platelet Count (10^3^/mm^3^)	306.26 ± 81.12	Cholesterol (mg/dL)	179.03 ± 34.52
WBCs (10^3^/mm^3^)	7.36 ± 2.59	Triglycerides (mg/dL)	145.71 ± 91.86
Random Blood Glucose (mg/dL)	98.9 ± 19.07	HDL-Cholesterol (mg/dL)	49.48 ± 15.15
ALT (U/L)	22.13 ± 12.09	LDL-Cholesterol (mg/dL)	103.96 ± 29.62

Data is presented as mean ± SD or frequency (percentage)

Abbreviations: ALT: Alanine Aminotransferase, ALP: Alkaline Phosphatase, AST: Aspartate Aminotransferase, BMI: Body Mass Index, Hb: Hemoglobin, Hc: Hematocrit, HDL: High Density Lipoprotein, LDL: Low Density Lipoprotein, RBCs: Red Blood Cells, WBCs: White Blood Cells

### Pharmacokinetics

**Fig. 2:**
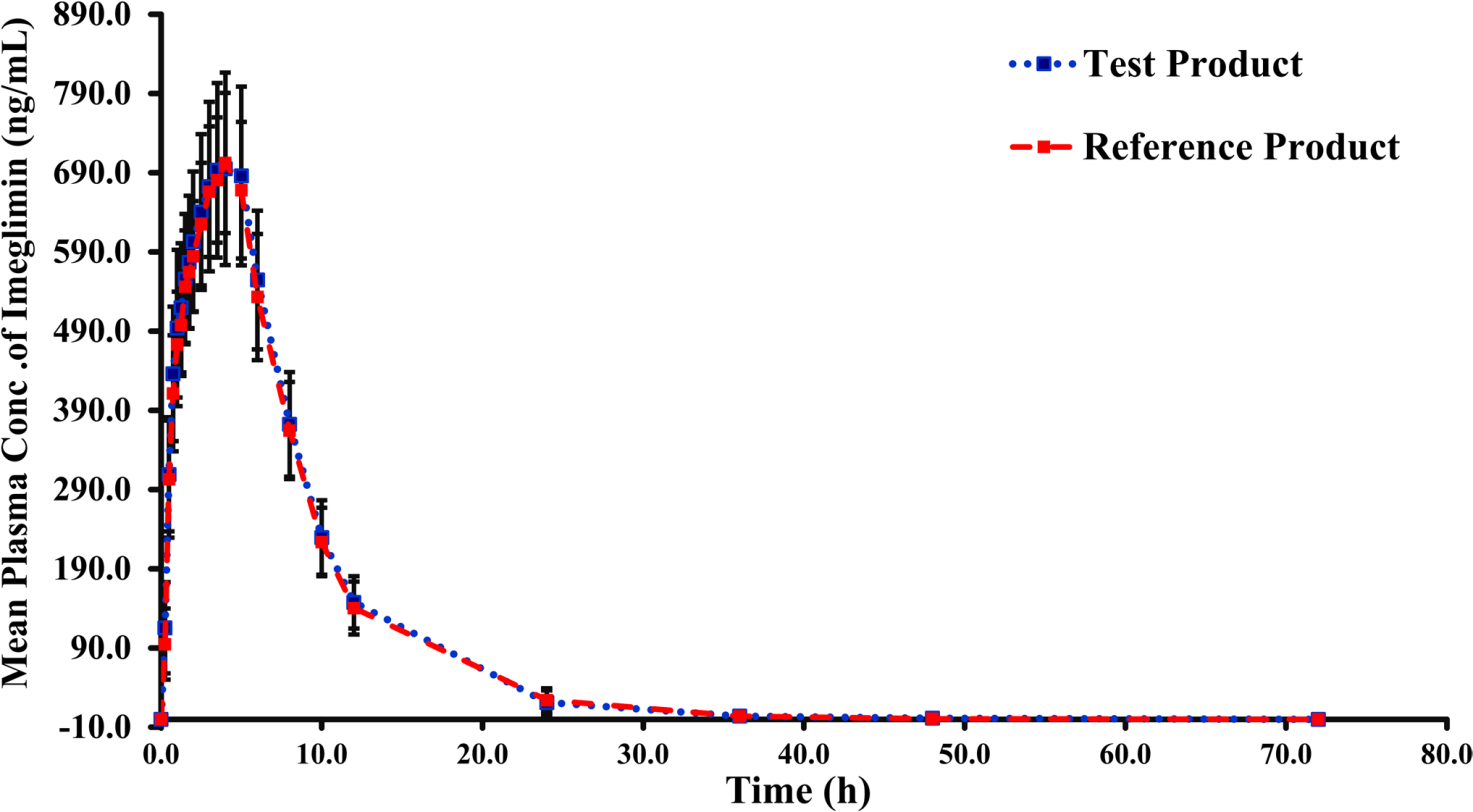
The mean plasma concentration–time profile of imeglimin hydrochloride 500 mg oral tablet following oral administration of one tablet of both Glimcoza (Test) and Twymeeg® (Reference) to healthy participants under fasting conditions

PK analysis was conducted for the included 29 subjects who completed the whole study periods and had adequate plasma concentration of imeglimin hydrochloride for evaluation. The mean plasma concentration–time curves for imeglimin hydrochloride following the oral administration of a single 500 mg oral tablet of imeglimin hydrochloride (Test) and Twymeeg® (Reference), under fasting conditions are illustrated in (Fig. 2). Following administration of the test and reference formulations to 29 healthy participants, it has been found that the average C_max_ of imeglimin hydrochloride was 783.53 ± 240.99 ng/mL for the test product and 751.52 ± 170.84 ng/mL for the reference product. The AUC_0–72_ was 6419.2 ± 2043.07 ng·h/mL and 6270.69 ± 1682.86 ng·h/mL and the AUC_0-inf_ was 6554.91 ± 2059.57 ng·h/mL and 6434.08 ± 1670.28 ng·h/mL for the test and reference products. The median T_max_ was 3.5 h for both products, with a range of (0.5–5) hours for the test product and (1–6) hours for reference product. The primary and secondary PK parameters for both products are listed in Table 2.

**Table 2:** Summary of pharmacokinetic parameters of imeglimin hydrochloride following administration of a single oral dose of glimcoza (Test) and Twymeeg® (Reference) tablet formulation in egyptian healthy subjects

PK Parameter, unit	Test (n = 29)	Reference (n = 29)
C_max_, ng/mL	783.53 ± 240.99	751.52 ± 170.84
C_last_, ng/mL	21.95 ± 12.15	26.102 ± 24.29
T_max_, h	3.5 (0.5–5)	3.5 (1–6)
AUC_0–72_, ng.h/mL	6419.201 ± 2043.07	6270.69 ± 1682.86
AUC_0-inf_ ng.h/mL	6554.91 ± 2059.57	6434.08 ± 1670.28
t_1/2_, h	4.51 ± 1.29	4.61 ± 1.22
K_e,_ h^−1^	0.166 ± 0.0513	0.163 ± 0.0544

Data are presented as mean ± SD, except for T_max_ which are median (range)

C_max_: the maximum plasma concentration, C_last_: the last quantifiable plasma concentration, T_max_: the time to reach maximum plasma concentration, AUC_0–72_: the area under the plasma concentration–time curve up to the 72 h, AUC_0-inf_ the area under the plasma concentration–time curve up to infinity, t_1/2:_ the elimination half-life, K_e_: elimination rate constant, PK: Pharmacokinetic

The mean AUC_₀–t_/AUC_₀–inf_ ratio exceeded the regulatory threshold of 80% for all participants in both the test and reference arms, indicating that the sampling duration was sufficient to capture the majority of systemic drug exposure (FDA 2004; EDA (2021); FDA (2024)). For the reference product, the mean ratio was 97.23% (range: 85.85–99.13%), while for the test product it was 97.71% (range: 93.82–99.23%), confirming minimal extrapolated AUC and compliance with BE guideline requirements for adequate characterization of the terminal elimination phase.

### Bioequivalence and statistical analysis

A summary of the BE evaluation of the test and reference products are shown in Table 3. The GMR% of the test to reference formulations for imeglimin hydrochloride, along with the corresponding 90% CI was 101.75% (94.42% – 109.64%) for C_max_, 100.66% (94.28% – 107.46%) for AUC_0–72,_ and 100.13% (93.77%—106.92%) for AUC_0-inf_. All these values fell within the predefined BE acceptance boundaries of 80–125% confirming the bioequivalence. The ISV coefficient of variation (CV%) for imeglimin hydrochloride was 14.69% for AUC_0–72,_ 14.74% for AUC_0-inf_ and 16.8% for C_max_. It has been shown that there was no statistically significant difference between treatments for logarithmically Ln-transformed C_max_, AUC_0–72_, AUC_0-inf_ (All at *p* > 0.05, respectively, ANOVA). There was no period, sequence or formulation effect on Ln (AUC_0–72_), Ln (AUC_0-inf_) and Ln (C_max_) (*p* > 0.05) indicating that there were no significant differences between the two formulations in AUC_0–72_, AUC_0-inf_ and C_max_ by two one-sided t-test. Using a nonparametric analysis (Wilcoxon signed-rank test), no statistically significant difference was found between the test and reference formulations in terms of T_max_, with a *p*-value of 0.128.

**Table 3:** Bioequivalence evaluation of imeglimin hydrochloride 500 mg after single oral dose administration of one tablet of test product (T) glimcoza versus reference product (R) twymeeg® under fasting conditions

PK Parameter	Geometric Means		GMR% Test/Reference	90% CI	P-value	CV_ws_%
	Test* Glimcoza	Reference* Twymeeg®				
C_max_ (ng/mL)	743.69	732.197	101.75	94.42–109.64	0.272	16.8%
AUC_0–72_ (ng.h/mL)	6059.404	6032.761	100.66	94.28–107.46	0.446	14.692%
AUC_0-inf_ (ng.h/mL)	6201.742	6207.213	100.13	93.77–106.92	0.536	14.74%

*N = 29 subjects

AUC_0–72_: the area under the plasma concentration–time curve up to the 72 h, AUC_0-inf_ the area under the plasma concentration–time curve up to infinity, C_max_: the maximum plasma concentration, CI: Confidence Interval, CVws: Within Subject Variability Coefficient of Variation, GMR%: Geometric Mean Ratio Percentage

### Safety evaluation

Throughout the whole study periods, all reported adverse events were mild in severity, drug-related, and no serious adverse events were experienced (Table 4). Only one participant experienced moderate decrease in platelet count. In the first period, one out of 29 participants (3.4%) reported hypotension as an adverse event during the administration of the test product, while in the second period, headache was reported by one participant (3.4%) after the administration of the test product, while two participants (6.89%) experienced headache and one participant (3.4%) reported abdominal pain after administration of the reference product. During the follow-up phase, CBC results revealed mild laboratory-related adverse events in some participants: low hemoglobin levels in 9 subjects (31.03%), reduced red blood cell count in 2 subjects (6.89%), moderate thrombocytopenia in 1 subject (3.4%), and mild leucopenia in 7 subjects (24.14%). As these laboratory findings were observed after the completion of both treatment periods, no causality could be attributed to a specific formulation. All vital signs remained within normal limits during both study periods.

**Table 4:** Summary of adverse events and laboratory findings

Type of Event	Formulation	Number of Participants (Frequency (%))	Severity
Summary			
No. of patients with moderate to severe AE	-	1 (3.4%, Moderate)	-
No. of patients with a SAE	-	None	-
Death	-	None	-
Clinical Adverse Events			
Hypotension	Test	1 (3.4%)	Mild
Headache	Test	1 (3.4%)	Mild
	Reference	2 (6.89%)	Mild
Abdominal pain	Reference	1 (3.4%)	Mild
Follow-up Laboratory Findings			
Low Hb	Both (follow-up)	9 (31.03%)	Mild
Reduced RBCs count	Both (follow-up)	2 (6.89%)	Mild
Thrombocytopenia	Both (follow-up)	1 (3.4%)	Moderate
Leukopenia	Both (follow-up)	7 (24.14%)	Mild

Abbreviations: *AE* adverse events, *Hb* hemoglobin, *RBC* red blood cell, *SAE* serious adverse event

## Discussion

To the best of our knowledge, this randomized, single-dose, two-period crossover trial is the first to demonstrate the BE between the test formulation of imeglimin hydrochloride 500 mg tablets (Glimcoza®) compared to the reference product (Twymeeg®) under fasting conditions in healthy adults in Egypt. Moreover, it represents the first PK evaluation of imeglimin conducted in a Middle Eastern and African population, providing important regional data to support its broader clinical use, thereby providing region-specific data that complement previously published studies in Japanese and Caucasian participants (Fouqueray et al. 2022).

The GMR and corresponding 90% CI for C_max_, AUC_0–72_, and AUC_0–inf_ were all within the regulatory acceptance boundaries of 80–125%, with relatively low ISV (14–17%), thereby fulfilling international criteria for BE (EDA (2021); FDA (2024)). Secondary PK parameter, including T_max_ (3.5 h), was also comparable between formulations, and safety evaluations confirmed that all reported adverse events were mild, with no serious adverse events observed. These findings confirm that both formulations exhibit comparable rates and extents of absorption, suggesting no excipient- or formulation-related impact on imeglimin disposition.

The PK results of this study are consistent with previously published data on imeglimin. Following a single 500 mg dose under fasting conditions, the observed median T_max_ of 3.5 h closely matched that reported in Caucasian volunteers (3.5 h) and was comparable to Japanese participants (3.0 h) in a phase 1 program (Fouqueray et al. 2022). The extent of exposure (AUC_0–inf_ ≈ 6,550 ng·h/mL) was within the range reported for both populations (6,279–6,631 ng·h/mL), while mean C_max_ values (~ 075–0.78 µg/mL) were slightly lower than the Caucasian mean (0.86 µg/mL) and below the Japanese mean (1.01 µg/mL), yet remained well within the published inter-subject variability (coefficient of variation (CV%) 16–25%). In addition, the elimination half-life estimated from our 72-h sampling window also fell within the reported 4.5–12 h in Japanese subjects, supporting the adequacy of exposure capture (Fouqueray et al. 2022). Importantly, the proportion of extrapolated AUC was < 5%, confirming that the 72-h sampling captured nearly the entire exposure profile, consistent with FDA guidance on BE study adequacy (FDA 2004; EDA (2021); FDA (2024)). Together, these findings support that the test and reference formulations exhibit PK profile consistent with global phase 1 data, with no formulation-related differences.

Importantly, all adverse events in our study were mild, with no serious events reported, in agreement with the favorable tolerability profile established in the TIMES clinical program (Dubourg et al. 2021b; Dubourg et al. 2021a; Dubourg et al. 2022; Reilhac et al. 2022). In TIMES-1, adverse event rates with imeglimin monotherapy were comparable to placebo, while TIMES-2 confirmed the long-term safety over 52 weeks, with gastrointestinal events being most frequently reported drug-related effects (FDA 2021b; Dubourg et al. 2022). TIMES-3 further demonstrated tolerability when imeglimin was combined with insulin, with hypoglycemic episodes limited to mild or moderate severity (Reilhac et al. 2022). In line with these findings, our observation of only mild and transient events (headache, abdominal pain) indicates that the generic formulation is well tolerated, does not introduce new or unexpected safety concerns and supports its equivalence to the reference product from both pharmacokinetic and tolerability perspectives.

The limited published data on imeglimin PK highlight the value of our findings. Most available data derive from Japanese or Western cohorts (Clémence et al. 2020; Chevalier et al. 2021; Fouqueray et al. 2022), with no data from Middle Eastern or African populations. By confirming BE in an Egyptian cohort, this study broadens the geographic evidence base and supports the global generalizability of imeglimin’s PK profile. Moreover, the results meet international regulatory standards, with 90% CIs for C_max_ and AUC contained within the accepted 80–125% range (FDA 2004; FDA (2024)). From both clinical and regulatory perspective, demonstrating BE under fasting conditions, not only ensures therapeutic interchangeability in line with regulatory guidance (FDA 2004; FDA (2024)), but also reinforces the reproducibility of imeglimin’s PK across diverse ethnic populations and formulations.

Importantly, confirmation of BE between the test and reference formulations has direct implications for access and policy. Although this study did not include a formal pharmacoeconomic assessment, the availability of a locally manufactured alternative is anticipated to improve affordability compared with the imported reference product. Future real-world studies may further evaluate the cost implications and potential health-system savings once the product becomes commercially available. Regulatory approval of the test formulation therefore represents an important step toward providing a cost-effective therapeutic option, particularly in resource-limited settings where treatment affordability remains a key barrier to optimal diabetes care (Giruzzi 2021). Expanding the availability of high-quality, locally produced generics has the potential to reduce treatment disparities and improve long-term glycemic outcomes.

## Conclusion

This randomized crossover trial confirmed the bioequivalence of test and reference imeglimin formulations in healthy Egyptian volunteers, with PK parameters fully meeting international regulatory criteria and only mild, transient adverse events reported. These results extend PK evidence to Middle Eastern and African populations and support wider access to affordable imeglimin therapy in Egypt and other resource-limited settings. Future research should evaluate the pharmacokineticsPK and safety of patients with T2D to further substantiate interchangeability in real-world clinical use.

Abbreviations: AE: Adverse event, Hb: Hemoglobin, RBCs: Red Blood Cells, SAE: Serious Adverse Event

## Data Availability

All data supporting the results reported in the manuscript are available upon request from the authors.
